# Preliminary evaluation of *ex vivo* and *in vivo* skin permeability of aromatic amines

**DOI:** 10.1371/journal.pone.0316150

**Published:** 2024-12-30

**Authors:** Yukie Yanagiba, Megumi Ono, Tatsushi Toyooka, Rui-Sheng Wang

**Affiliations:** Research Center for Chemical Information and Management, National Institute of Occupational Safety and Health, Kawasaki, Japan; University Hospital of Padova, ITALY

## Abstract

A potential link has been reported between skin exposure to aromatic amines, such as ortho-toluidine (OT) and 3,3′-dichloro-4,4′-diaminodiphenylmethane (MOCA), and bladder cancer cases observed in Japanese chemical factories. To evaluate this association, we explored the permeability of OT and MOCA through pig skin and investigated the subsequent changes in plasma and urine concentrations in rats following percutaneous exposure. Employing Yucatan micropig skin, we first executed a permeability test by affixing the skin to a diffusion cell and applying ^14^C-labeled OT or MOCA. The receptor fluid’s radioactivity was quantified at intervals of 1, 3, 6, 8, 24, and 48 h after application using a liquid scintillation counter. Next, we applied lint cloths drenched in OT and MOCA solutions to the backs of 7-week-old male F344 rats and monitored plasma and urine concentrations over time. Additionally, we investigated the pharmacokinetics of ^14^C-labeled OT or MOCA solutions for 8 h following percutaneous administration. Both OT and MOCA demonstrated high skin penetration; in particular, plasma concentrations significantly rose at 6 h for OT and 8 h for MOCA after exposure. However, OT was rapidly absorbed into the bloodstream and swiftly excreted into the urine, indicating quick absorbability. In contrast, MOCA penetrated the skin quickly but exhibited delayed bloodstream entry and urinary excretion, suggesting slower absorbability. Pharmacokinetic findings revealed the rapid urinary excretion of OT, whereas MOCA was excreted in the urine and potentially in the feces as well via bile. These findings indicate that implementing measures based on chemical absorbability could significantly enhance the management of industrial chemicals where percutaneous absorbability is a concern.

## Introduction

Occupational bladder cancer cases have been increasingly emerging among workers handling aromatic amines like ortho-toluidine (OT) in Japanese chemical factories, posing a significant societal health issue. Despite controlled and permissible concentration conditions, the dermal absorption of these chemicals has been implicated in health problems, drawing increased scrutiny toward percutaneous exposure to industrial chemicals. Notably, OT was classified as a Group 1 carcinogen by the International Agency for Research on Cancer (IARC) in 2012, highlighting its carcinogenic potential in humans. An investigation into exposure routes, particularly for OT, revealed that despite workers wearing dust masks, rubber gloves, and protective clothing, and OT concentrations in the workplace being below the permissible concentration (0.003 ppm compared to 1 ppm), high concentrations of OT and its metabolites were detected in the urine of workers, which could not be explained by inhalation exposure [1]. This was further supported by findings of OT contamination inside rubber gloves; moreover, interviews showed that workers were required to wear short-sleeved clothing during the summer. Thus, a considerable amount of OT was reported to have been absorbed through percutaneous exposure [2]. Additionally, as with OT, 3′,3′-dichloro-4,4′-diaminodiphenylamine, MOCA, classified as a Group 1 carcinogen by the IARC in 2010, has been linked to percutaneous exposure in bladder cancer cases. Reports have also indicated that the urinary MOCA concentration of a 30-year-old male worker who was exclusively exposed to a MOCA solution on his upper body reached its peak level at 4 h post-exposure, and that low MOCA concentrations were detected in the urine over the next 4 days [3]. A feature of MOCA was accordingly inferred to be its propensity to accumulate in the body.

These findings underscore the critical role of percutaneous exposure in occupational chemical hazards for substances like OT and MOCA. However, the current lack of scientific evidence on the skin absorbability, permeability, and physicochemical characteristics of these chemicals highlights a gap in our understanding necessary for risk assessment. Thus, in this study, we aimed to investigate the skin permeability of OT and MOCA, which has become problematic in Japan, using pig skin and tracking changes in *in vivo* plasma and urine concentrations over time. Additionally, in this context, penetration denotes the entry of chemicals into the skin, permeation denotes their passage through the skin, and absorption denotes their entry from the skin into the bloodstream [4].

## Materials and methods

### Chemical substances

We used ^14^C-labeled OT and MOCA for *ex vivo* skin permeability experiments. OT and MOCA were synthesized by American Radiolabeled Chemicals Inc. (Saint Louis, MO, USA) and BlyChem Ltd. (Billingham, UK), respectively. The OT (≥99.5%) and 3,3′-dichlorobenzidine used in the *in vivo* percutaneous exposure experiment were purchased from Sigma Aldrich Japan (Tokyo, Japan), the MOCA (≥89.5%) used in the *in vivo* experiment was purchased from Tokyo Chemical Industry Co., Ltd. (Tokyo, Japan), and acetone (reagent grade) was purchased from Fujifilm Wako Pure Chemical Corp. (Tokyo, Japan). Methanol (for LC-MS) and formic acid (reagent grade) were purchased from Fujifilm Wako Pure Chemical Corp. (Tokyo, Japan) for the analysis of plasma and urine samples.

### *Ex vivo* skin permeability experiment

Skin collected from Yucatan micropigs (YMP) was used for skin permeability experiments. YMP skin was collected from a 5-month-old YMP, and the skin set that was cryopreserved at −80°C was purchased from Charles River Laboratories Japan, Inc. (Yokohama, Japan), with the skin kept at −80°C until use. The frozen skin (back) was thawed naturally at room temperature (20°C), the subcutaneous fat and meat pieces were removed with scissors, and the fat attached to the dermis was removed using a file. A vertical glass diffusion cell (effective area of 1.8 cm^2^) was used for the skin permeability experiments. Skin integrity was checked with an ohm meter (Digital Multimeter DMM6000, ASONE Corporation, Japan). Skin resistance above 50 kΩ was considered intact, according to previous studies [5]. Tissue specimens were also prepared from a portion of the skin used in the experiment, and the skin was observed after hematoxylin and eosin staining to ensure that there was no damage. Approximately 9.5 mL of phosphate-buffered saline (PBS) supplemented with kanamycin was kept at 33°C and stirred in the receptor phase of the vertical diffusion cell. We added 0.2 mL of the sample to the donor phase, and 0.2 mL of the receptor phase was collected at 1, 3, 6, and 24 h after sample addition, with the radioactivity of the receptor fluid collected every hour and measured using a liquid scintillation counter. We also conducted similar measurements of skin radioactivity 3 h later to confirm skin penetration. For the spiked samples, ^14^C-labeled OT and MOCA were each dissolved in PBS to a concentration of 0.1 μCi and used in the experiment. The skin permeation rate was calculated by dividing the radioactivity of the receptor layer at each sampling time by the radioactivity of the added test substance. The normalized cumulative amount (% dose applied/cm^2^) was used for the Figure.

### Percutaneous exposure experiment

Experiment (1): For the percutaneous exposure experiment, we used male F344 rats (n = 3) purchased from Charles River Laboratories Japan, Inc. and acclimatized them for 1 week until the start of the experiment (7 weeks old at the start of the experiment). The backs of the rats were sheared and shaved, after which they underwent percutaneous exposure to OT (100 mg/kg body weight) or MOCA (100 mg/kg body weight (bw)) for 24 h using a lint cloth (3 × 3 cm). Three rats were used in each group, with 80% acetone in PBS as the solvent. Acetone, which has been reported to not affect the lipid composition of the stratum corneum, was selected as the solvent [6, 7]. In another experiment, the condition of the skin after 24-hour dermal administration of acetone was observed histologically, and it was confirmed that no damage was observed (unpublished data). Blood was sampled from the tail at 1, 3, 6, 8, 24, 48, and 72 h after the start of exposure. The lint cloth on the back was removed 24 h after the start of the exposure. Urine was collected from the metabolic cages at the start of exposure. The urine collection intervals were 0–24, 24–48, and 48–72 h. This study was carried out in strict accordance with the recommendations in the Guide for the Laboratory Animal Committee of the National Institute of Occupational Safety and Health, Japan. The protocol was approved by the Committee on the Ethics of Animal Experiments of the National Institute of Occupational Safety and Health, Japan (Protocol Number: R01-01-2). All surgery was performed under an isoflurane inhalation anesthetic solution, and all efforts were made to minimize suffering.

Experiment (2): We used male Sprague-Dawley rats (7 weeks old) to confirm the pharmacokinetics. We used male Sprague-Dawley rats purchased from Charles River Laboratories Japan, Inc. and acclimatized them for 1 week before the experiment began (7 weeks old at the start of the experiment). The administered solution was prepared by dissolving ^14^C-labeled OT and MOCA and unlabeled OT or MOCA in a 60% acetone-PBS solution and adjusting the concentration to be 12.5 mg/185 kBq/mL, respectively. After shearing and shaving the backs of the rats, 4 mL of the test substance was added to a 3 x 3 cm lint cloth and applied for percutaneous exposure. After the end of administration, the lint cloth was removed, and the administered solution remaining at the administered site was wiped off. Further, the animals were euthanized by excess carbon dioxide inhalation under isoflurane inhalation anesthesia, 40-μm-thick sections were prepared on three sides (left side, left paramedian side, and median side) for each animal, and whole-body autoradiography was conducted. Rats in the OT treatment group (n = 1) were housed in a metabolic cage from the start of treatment, and urine was collected up to 24 h later; rats in the MOCA treatment group (n = 1) underwent the same procedure. A portion of the urine (200 μL) was then used for radioactivity measurements.

### LC-MS/MS analysis method

LC-MS/MS was used to analyze plasma and urine samples. The plasma was mixed in a 1:1 ratio with a 4% aqueous phosphoric acid solution. The urine was centrifuged at 1,000 rpm, the supernatant was mixed in a 1:1 ratio with water, and solid-phase extraction was conducted using Oasis PRiME HLB, the result of which was used as the analysis sample. 3,3′-dichlorobenzidine was used as the internal standard for analysis.

AcQuity H CLASS (Nippon Waters, Tokyo, Japan) was used for liquid chromatography and ACQUITY BEH C18, 1.7 μm, 2.1 mm × 50 mm (Waters, Tokyo, Japan) was used for column chromatography. The mobile phases used were A) water + 0.1% formic acid and B) methanol + 0.1% formic acid. A tandem quadrupole mass spectrometer ZEVO TQD was used as the detector (Nippon Waters, Tokyo, Japan). The analysis conditions were as follows: Ionization mode: ESI+, capillary voltage: 0.8 kV, probe temp: 450°C, acquisition, selected ion recording (SIR), and cone voltage: 15 V. The m/z values of OT and MOCA were 108 and 267 for MOCA.

### Statistical analysis

Statistical analysis was performed using the GraphPad Prism 8 software (GraphPad Software, San Diego, CA, USA). All data are shown as the mean ± standard deviation (SD). Comparisons were made using 1-way ANOVA, with a value of p < 0.05 considered statistically significant. The first sampling time after administration and between each sampling time point was evaluated using Dunnett’s multiple comparison test. All experiments in this study incorporated within-subjects design.

## Results

### *Ex vivo* skin permeability experiment

**Fig 1:**
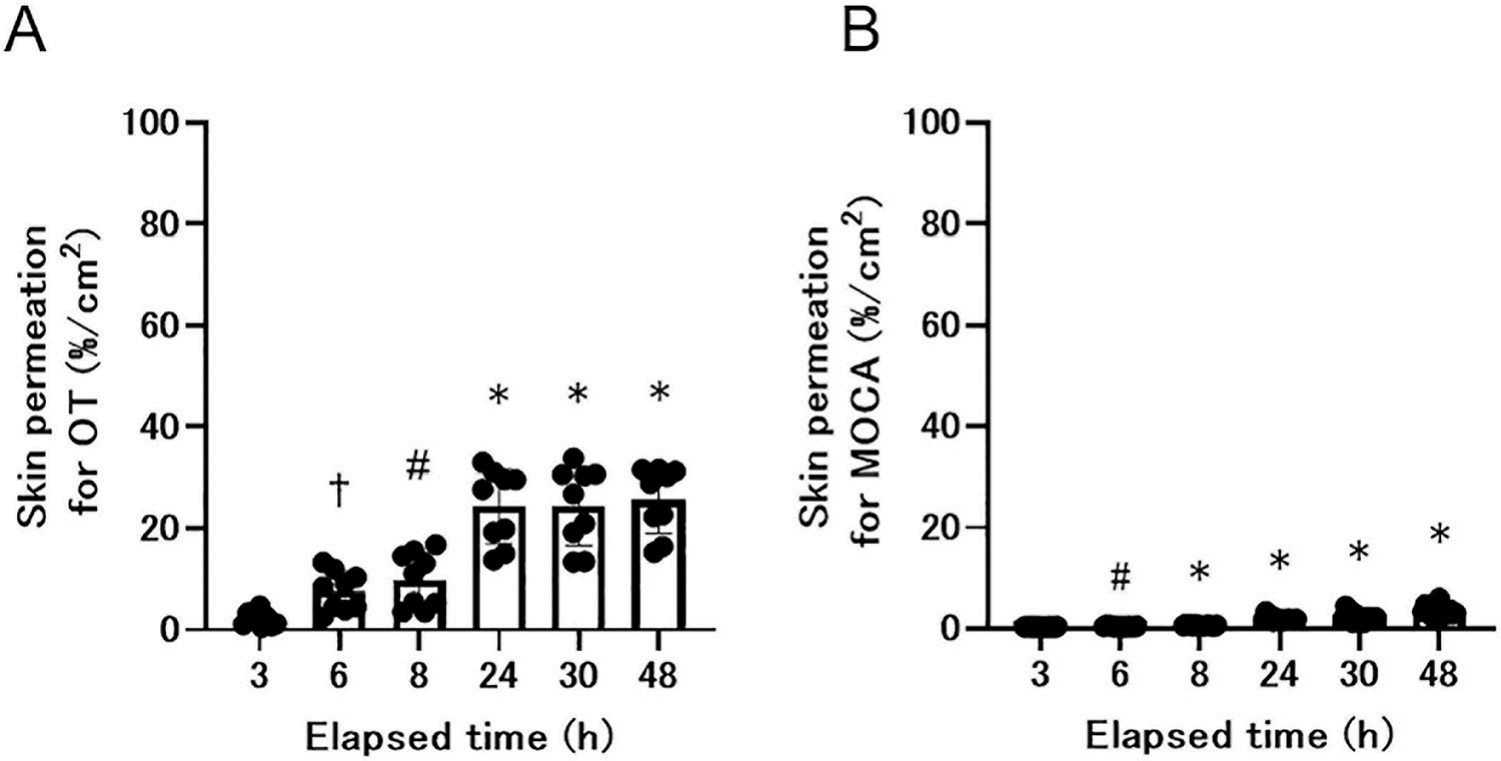
Pig skin permeation rate for each test substance. A. Pig skin permeation for OT, B. Pig skin permeation for MOCA. Each value represents the mean and SD. Individual data points are shown as dots. Significant differences relative to the values at 3 h are indicated by †p < 0.01, #p < 0.001, and *p < 0.0001. Statistical analysis was performed using Dunnett’s test.

Fig 1 shows the pig skin permeation rates for each test substance. The skin permeation values for OT were 2.2%/cm^2^ ± 1.4%/cm^2^ at 3 h after addition, 9.9%/cm^2^ ± 5.4%/cm^2^ after 8 h, and 24.4%/cm^2^ ± 7.3%/cm^2^ after 24 h. For MOCA, the values were 0.4%/cm^2^ ± 0.14%/cm^2^ after 3 h, 0.7%/cm^2^ ± 0.1%/cm^2^ after 8 h, and 2.0%/cm^2^ ± 0.5%/cm^2^ after 24 h. We also confirmed that the values in the skin at 3 h after addition were 14.5%/cm^2^ ± 1.0%/cm^2^ for OT and 32.1%/cm^2^ ± 1.6%/cm^2^ for MOCA.

### Changes in plasma and urine concentration after percutaneous exposure

**Fig 2:**
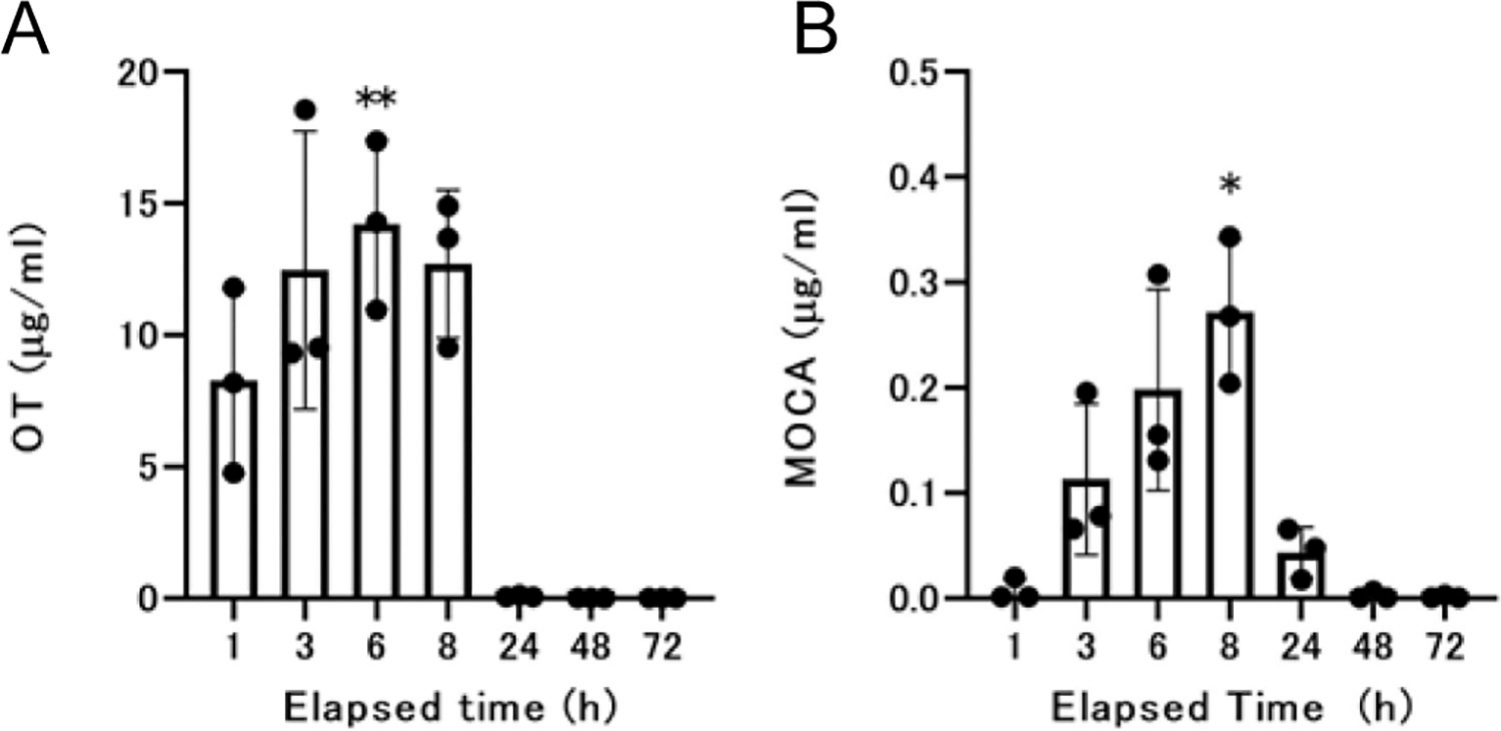
Concentration of OT or MOCA in plasma. The plot shows the concentrations of OT or MOCA in plasma. A. Concentrations of OT in plasma, B. concentrations of MOCA in plasma. Arithmetic means and SDs are presented. Each value represents the mean and SD. Individual data are also shown as dots. Significant differences relative to the concentrations at 1 h are shown by *p < 0.05 and **p < 0.01. The statistical analysis was performed using Dunnett’s test.

**Fig 3:**
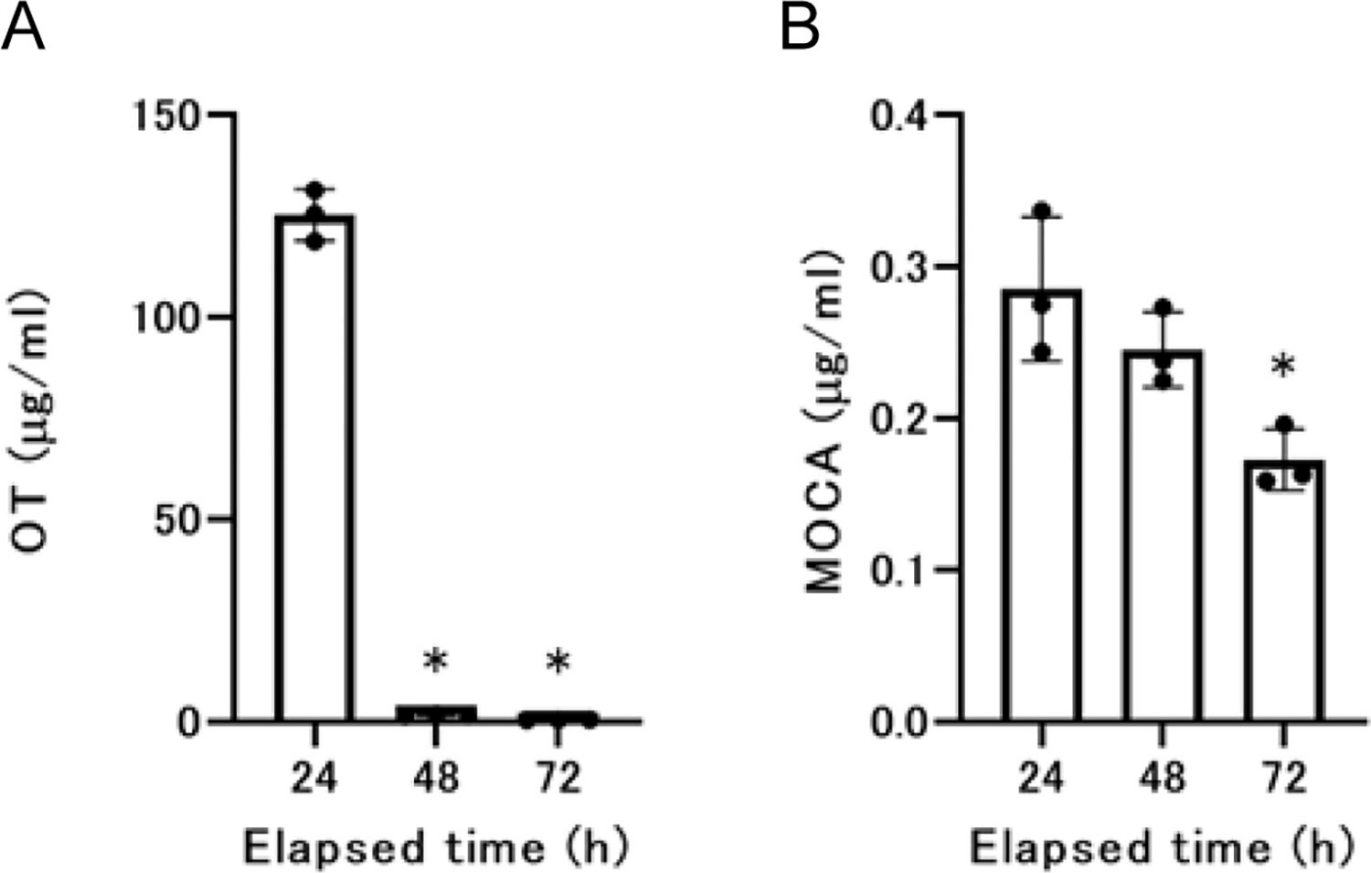
Concentration of OT or MOCA in urine. The plot shows the concentrations of OT or MOCA in urine. A. Concentrations of OT in urine, B. concentrations of MOCA in urine. Arithmetic means and SDs are presented. Each value represents the mean and SD. Individual data are also shown as dots. Significant differences relative to the concentrations at 24 h are shown by *p < 0.05 and **p < 0.01. The statistical analysis was performed using Dunnett’s test.

We used rats to observe the changes in the plasma and urine concentrations of OT and MOCA after percutaneous exposure. The time of significantly higher plasma concentrations compared to the initial sampling time was 6 h for OT and 8 h for MOCA (Fig 2). We also confirmed from an analysis of urine concentrations after percutaneous exposure that OT was rapidly excreted into the urine but that the urinary excretion of MOCA was slow or low (Fig 3). The excretion rates calculated from urinary radiation levels up to 24 h after the start of administration were 81.3% for OT and 5.4% for MOCA.

### *In vivo* distribution

**Fig 4:**
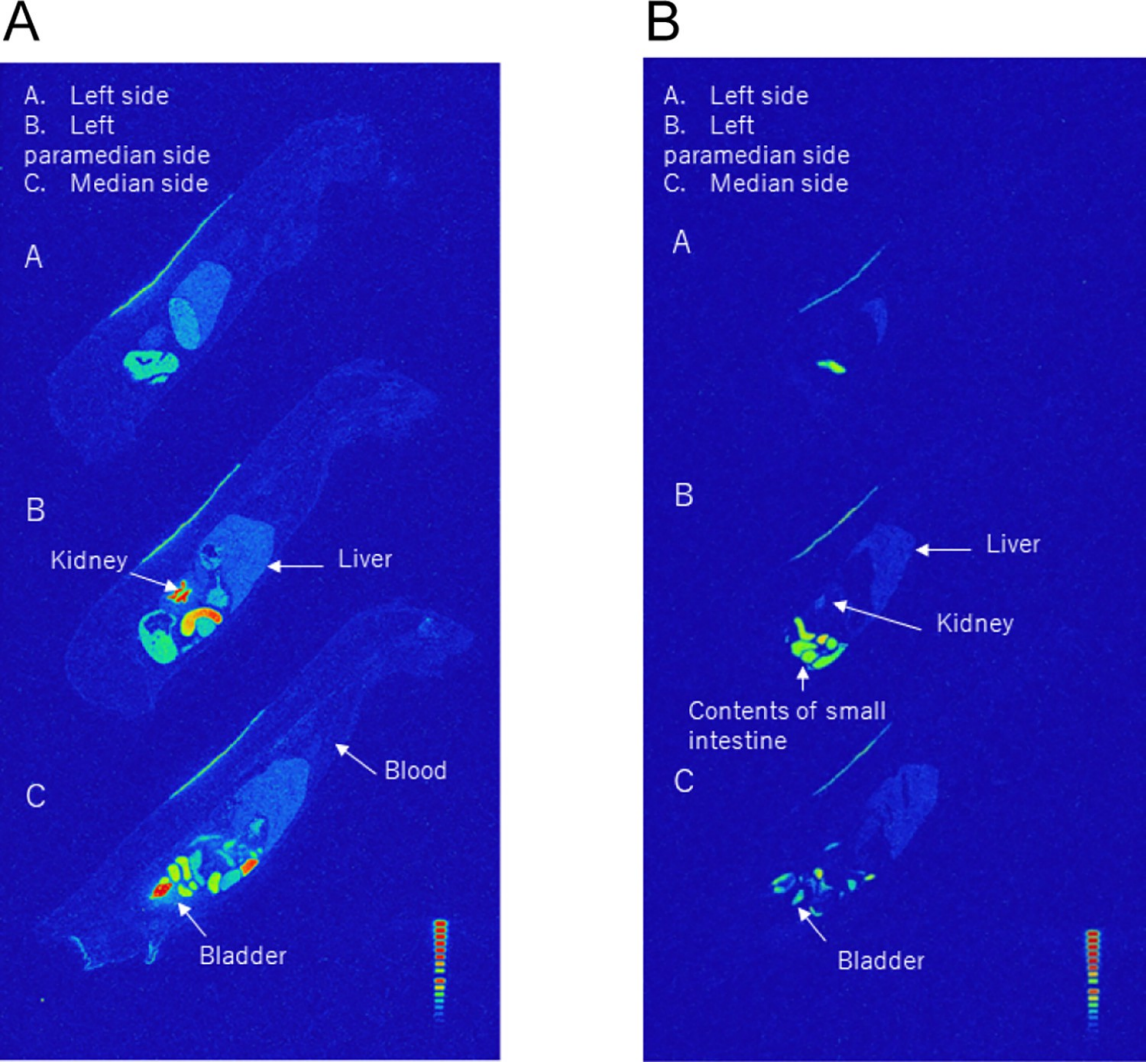
Pharmacokinetics after percutaneous exposure. A. Eight hours after the start of OT administration (0 h after the end of administration) at 50 mg/517 kBq/4 mL/kg bw. B. Eight hours after the start of MOCA administration (0 h after the end of administration) at 50 mg/517 kBq/4 mL/kg bw.

Whole-body autoradiography results confirmed extremely high radiation in the bladder 8 h after exposure to OT, and its rapid excretion into the urine was observed (Fig 4A). Radiation signals in the tissues of MOCA were observed. Signals were also observed in the bladder, but not as strong as those of OT. Alternatively, for MOCA, radiation in the bladder was not elevated; therefore, urinary excretion was slow or minimal. However, high radiation was observed in the small intestine (Fig 4B).

## Discussion

Both OT and MOCA exhibit high skin penetrability, yet they differ significantly in terms of skin permeability and absorbability. OT rapidly enters the bloodstream following skin penetration and is quickly excreted in the urine, demonstrating fast skin absorbability. Conversely, despite MOCA’s rapid skin penetration, it takes longer to enter the bloodstream and be excreted in the urine, indicating slower skin absorbability.

When the skin absorbs a chemical, the substance partitions into the skin before penetrating and absorbing deeply. This partitioned chemical also diffuses through the skin matrix, moving from areas of higher concentration to those with lower concentration. As a result, chemicals on the skin’s surface gradually migrate deeper, with certain substances being absorbed by capillaries and entering the general circulation [4]. Compounds with an octanol-water partition coefficient (log*K*_ow_) of approximately 2 are considered to be readily absorbed by the skin [8]. The stratum corneum, the skin’s outermost lipophilic layer, serves as the primary barrier to chemical absorption [9]. Consequently, a substance’s skin permeation increases with its lipophilicity or log*K*_ow_; however, permeation decreases when log*K*_ow_ exceeds 3 [10, 11]. For instance, methyl paraben, with a log*K*_ow_ of 1.93, shows higher permeability than n-propyl paraben, which has a log*K*_ow_ of 2.81, whereas n-butyl paraben, with a log*K*_ow_ of 3.53 and n-pentyl-paraben, with a log*K*_ow_ of 4.1, exhibit reduced permeability, especially in skin lacking the stratum corneum compared to full-thickness skin. Substances with a high log*K*_ow_ act as a permeation barrier due to their low diffusivity in the epidermis and dermis [11]. Furthermore, the skin contains metabolic enzymes (albeit not as extensively as the liver), allowing for metabolism within the skin itself [12, 13].

Drug-metabolizing enzymes involved in the metabolism of chemicals, such as phase 1 enzymes CYP450 and phase 2 conjugating enzymes, also exist in the skin. The activity of the phase 1 enzyme CYP450 in the skin, as measured by marker substrate metabolism, is reported to be 0%–27% of that in the liver, whereas the activity of some phase 2 conjugating enzymes is 0%–50% of that in the liver [10, 14].

In our experiments, we used the entire skin without exfoliation. Therefore, MOCA, which has a log*K*_ow_ of 3.9, is thought to quickly penetrate the stratum corneum and diffuse into the skin over time. However, owing to the high lipophilicity of MOCA, diffusion below the stratum corneum (epidermis and dermis) may be slow, resulting in lower skin permeability. Compounds with a log*K*_ow_ of 2 or more have been shown to accumulate in the fat compartment of the dermis. Although bisphenol-A (BPA), which has a log*K*_ow_ of 3.1, has high lipophilicity, it is thought to have fast skin penetration but high levels of accumulation in the dermis [15]. MOCA, which has a log*K*_ow_ similar to that of BPA, is speculated to accumulate in the dermal compartment like BPA. Furthermore, the radiation dose was observed to be higher in the contents of the small intestine than in the bladder’s urine, suggesting that MOCA, once in the blood, may be metabolized in the liver, conjugated, and then excreted via bile. Meanwhile, OT, with a log*K*_ow_ of 1.32, exhibits slow penetration into the stratum corneum but is believed to diffuse into the lower layers (epidermis and dermis) due to its lesser lipophilicity compared to that of MOCA. Thus, OT, which easily enters the blood, might be rapidly excreted into the urine. Additionally, the possibility exists for unmetabolized OT in the urine as percutaneous administration bypasses the first metabolic pass. The stratum corneum, along with the epidermis and dermis, serves as a barrier to the skin permeation of lipophilic chemicals, indicating that differences in log*K*_ow_ could significantly impact intradermal behavior and pharmacokinetics. Biological monitoring is a method for exposure assessment that is particularly effective for OT, which is rapidly excreted into the urine after skin permeation by measuring post-work urine concentration. However, this study focused solely on free MOCA without analyzing urinary metabolites. Future analysis should include metabolites. Concerns with MOCA involve its fast skin penetration but slow skin permeation, low urinary excretion, and potential accumulation in the dermal compartment. For exposure assessments, it is essential to use hydrolyzed MOCA (total-MOCA) as an index and continuously monitor urine concentration beyond the end of work.

Occupational percutaneous exposure plays a significant role in workers’ total exposure, occurring under various conditions and with all types of chemicals. Although the importance of the dermal route in occupational environments is recognized, specific assessment methods for skin exposure remain undefined. The United States Occupational Safety and Health Administration (OSHA) standards for 4,4′-methylenedianiline acknowledge skin exposure as a major exposure route for workers but have limited guidance on exposure levels [16, 17]. Moreover, despite proposals to lower airborne exposure limits based on new toxicity information and improved risk assessment methods, the significance of the dermal route increases without parallel efforts to reduce percutaneous exposure. For instance, OSHA estimates that reducing airborne benzene concentration from 10 to 1 ppm could raise skin uptake from 4% to 30% of daily exposure among workers engaged in tire manufacturing in the United States [17]. Therefore, integrating airborne and percutaneous exposures is crucial for accurately assessing workplace hazards and enhancing worker protection.

This study represents an initial evaluation of OT and MOCA’s skin permeability with a limited sample size. Future research should include a larger sample size. However, gathering experimental toxicity information on chemical substances’ skin absorbability is time-consuming. Whereas advances in *in silico* methods, such as structure-activity relationships, have been made, their application in occupational health is not yet widespread. Thus, in industrial settings, taking measures based on estimated skin absorbability using the log*K*_ow_ value is an effective strategy for managing chemicals and preventing health issues among workers. It is also necessary to develop sampling times and indicators suitable for future exposure assessment based on skin absorbability.

## References

[1] Ministry of Health, Labour and Welfare of Japan. Disaster survey regarding bladder cancer that occurred in chemical factory in Fukui Prefecture; 2016. [Cited 2023 August 29]. Available from: https://www.mhlw.go.jp/file/04-Houdouhappyou-11305000-Roudoukijunkyokuanzeneiseibu-Kagakubushitsutaisakuka/0000126164.pdf.

[2] Nakano M, Omae K, Takebayashi T, Tanaka S, Koda S. An epidemic of bladder cancer: ten cases of bladder cancer in male Japanese workers exposed to Ortho-toluidine. J Occup Health. 2018;60: 307–311. doi: 10.1539/joh.2017-0220-OA 29743389 PMC6078838

[3] Osorio AM, Clapp D, Ward E, Wilson HK, Cocker J. Biological monitoring of a worker acutely exposed to MBOCA. Am J Ind Med. 1990;18: 577–589. doi: 10.1002/ajim.4700180508 2244630

[4] Sugibayashi K. Theory, practical application and future expectation of percutaneous absorption. Oleoscience. 2017;17: 549–558. doi: 10.5650/oleoscience.17.549

[5] Schenk L, Rauma M, Fransson MN, Johanson G. Percutaneous absorption of thirty-eight organic solvents in vitro using pig skin. PLOS ONE. 2018;13: e0205458. doi: 10.1371/journal.pone.0205458 , PMCID: PMC6209206. 30379962 PMC6209206

[6] Barba C, Alonso C, Martí M, Manich A, Coderch L. Skin barrier modification with organic solvents. Biochim Biophys Acta. 2016;1858: 1935–1943. doi: 10.1016/j.bbamem.2016.05.009 [Epub 2016 May 14]. . 27184268

[7] Barba C, Alonso C, Martí M, Carrer V, Yousef I, Coderch L. Selective modification of skin barrier lipids. J Pharm Biomed Anal. 2019;172: 94–102. doi: 10.1016/j.jpba.2019.04.040 [Epub 2019 Apr 19]. . 31029804

[8] Armette Wilschut W. FtB, Peter J. Robinson, Thomas E. McKone. Estimating skin permeation. The validation of five mathematical skin permeation models. Chemosphere. 1995;30: 1277–1296. 10.1016/0045-6535(95)00023-2 7749723

[9] Herrero M, Rovira J, Esplugas R, Nadal M, Domingo JL. Human exposure to trace elements, aromatic amines and formaldehyde in swimsuits: assessment of the health risks. Environ Res. 2020;181: 108951. doi: 10.1016/j.envres.2019.108951 [Epub 2019 Nov 22]. . 31784079

[10] Poet TS, McDougal JN. Skin absorption and human risk assessment. Chem Biol Interact. 2002;140: 19–34. doi: 10.1016/s0009-2797(02)00013-3 12044558

[11] Oshizaka T, Todo H, Sugibayashi K. Effect of direction (epidermis-to-dermis and dermis-to-epidermis) on the permeation of several chemical compounds through full-thickness skin and stripped skin. Pharm Res. 2012;29: 2477–2488. doi: 10.1007/s11095-012-0777-6 22622509

[12] Yamaguchi K, Mitsui T, Aso Y, Sugibayashi K. Structure-permeability relationship analysis of the permeation barrier properties of the stratum corneum and viable epidermis/dermis of rat skin. J Pharm Sci. 2008;97: 4391–4403. doi: 10.1002/jps.21330 18228598

[13] Boogaard PJ, Denneman MA, Van Sittert NJ. Dermal penetration and metabolism of five glycidyl ethers in human, rat and mouse skin. Xenobiotica. 2000;30: 469–483. doi: 10.1080/004982500237488 10875681

[14] Baron JM, Höller D, Schiffer R, Frankenberg S, Neis M, Merk HF, et al. Expression of multiple cytochrome P450 enzymes and multidrug resistance-associated transport proteins in human skin keratinocytes. J Invest Dermatol. 2001;116: 541–548. doi: 10.1046/j.1523-1747.2001.01298.x 11286621

[15] Kaddar N, Harthé C, Déchaud H, Mappus E, Pugeat M. Cutaneous penetration of bisphenol A in pig skin. J Toxicol Environ Health A. 2008;71: 471–473. doi: 10.1080/15287390801906824 . 18338280

[16] Bos PM, Brouwer DH, Stevenson H, Boogaard PJ, de Kort WL, van Hemmen JJ. Proposal for the assessment of quantitative dermal exposure limits in occupational environments: Part 1. Development of a concept to derive a quantitative dermal occupational exposure limit. Occup Environ Med. 1998;55: 795–804. doi: 10.1136/oem.55.12.795 9924440 PMC1757540

[17] Fenske RA, van Hemmen JJ. Occupational skin exposure to chemical substances: setting limits. Ann Occup Hyg. 1994;38: 333–336. doi: 10.1093/annhyg/38.4.333-a . 7978958

